# Parent‐ and child‐driven effects during the transition to adolescence: a longitudinal, genetic analysis of the home environment

**DOI:** 10.1111/desc.12432

**Published:** 2016-06-19

**Authors:** Laurie J. Hannigan, Tom A. McAdams, Robert Plomin, Thalia C. Eley

**Affiliations:** ^1^ King's College London UK

## Abstract

Theoretical models of child development typically consider the home environment as a product of bidirectional effects, with parent‐ and child‐driven processes operating interdependently. However, the developmental structure of these processes during the transition from childhood to adolescence has not been well studied. In this study we used longitudinal genetic analyses of data from 6646 UK‐representative twin pairs (aged 9–16 years) to investigate stability and change in parenting and household chaos in the context of parent–child bidirectional effects. Stability in the home environment was modest, arising mainly from parent‐driven processes and family‐wide influences. In contrast, change over time was more influenced by child‐driven processes, indicated by significant age‐specific genetic influences. Interpretations of these results and their implications for researchers are discussed.

## Research highlights

- Parent‐driven processes and family‐wide factors are found to exert predominantly stable influences on children's perceptions of their home environment as they enter adolescence.
- Child‐driven processes, indicated by the influence of children's genes, operate primarily age‐specifically and are involved in driving change in perceived parenting and household chaos across this period.
- Evidence of age‐specific child‐driven processes illustrate the effects of behavioural and perceptual change on children's experiences of their home environment in early adolescence.

## Introduction

The home environment has long been a central focus of study in child development. It is a broad concept, encompassing the range of processes and relationships that exist within a household (Bronfenbrenner, 1977, 1979; Sameroff, 1986). Taken together, these factors provide the primary context for child and adolescent development, and their significance is highlighted by extensive evidence of associations with behavioural, social and emotional outcomes (Bradley, Corwyn, Burchinal, McAdoo & García Coll, 2001a; Bradley, Caldwell, Rock, Ramey, Barnard *et al*., 1989; Evans, 2006; Rutter, 1985a, 1985b). However, the home environment is not *only* contextual. It is also shaped by the child, whose traits and behaviours elicit responses from those around them in the household (Bell, 1979; Scarr & McCartney, 1983). The main goal of this research is to investigate the developmental structure of these child‐driven processes, relative to that of parent‐driven processes and family‐wide factors, as they shape different aspects of the home environment during the transitional period between childhood and adolescence.

### The home environment in child development: context and consequence

Many theoretical accounts of child development advocate considering the home environment as a collection of reciprocal processes and relationships (e.g. Bell, 1968, 1979; Cook & Kenny, 2005; Kenny & La Voie, 1984; Sameroff, 1975). This means that, instead of looking only at how aspects of the home environment can influence a developing child, attention must also be paid to how the characteristics and behaviour of the child may influence his or her home environment (Collins, Maccoby, Steinberg, Hetherington & Bornstein, 2000; Lollis & Kuczynski, 1997; Maccoby, 2000). Given that parents (and their parenting behaviours) represent such a central component of this environment, this kind of model is often operationalized in terms of *bidirectional effects* (e.g. Alemany, Rijsdijk, Haworth, Fañanás & Plomin, 2013; Larsson, Viding, Rijsdijk & Plomin, 2008). *Bidirectionality*, therefore, describes the way in which child‐driven and parent‐driven processes combine to shape the home environment.

Theories that emphasize the need to consider child‐driven processes in the home environment are supported by consistent findings, from several decades of behavioural genetic research, which show that putatively environmental measures are subject to the influence of children's genes (Kendler & Baker, 2007; Jaffee & Price, 2007). A recent meta‐analysis of genetically sensitive, children‐as‐twin studies of parenting found that more than one‐fifth (23%) of the variance in parenting was associated with children's genes (Avinun & Knafo, 2014). In studies of this kind, genetic influence on parenting can be interpreted as evidence of the effects of children's genetically influenced behaviour on the parenting that they receive (Plomin & Bergeman, 1991; Scarr & McCartney, 1983). Associations between children's genotypes and environments that are mediated in this manner are known as *evocative* gene–environment correlations (Plomin, DeFries & Loehlin, 1977). This term connotes the role of child behaviours in *evoking* a response from the environment – in this case, via their parents. The concept of evocative gene–environment correlation aligns with the child‐driven processes described in bidirectional models of parenting and child development (Collins *et al*., 2000). Consequently, behavioural genetic designs able to detect gene–environment correlations offer a useful test of the extent to which aspects of the home environment can be considered as truly *environmental* contexts for child development (Klahr & Burt, 2014).

The inherent potential of behavioural genetic designs for investigating the relative importance of parent‐ and child‐driven processes makes them an important tool in developmental research. In one respect, this is because accounting for genetic influence on putative environmental measures is necessary to distinguish potentially causal associations with child developmental outcomes from those that arise as a result of shared genes (Jaffee, Strait & Odgers, 2012). Crucially though, this is also because the processes of gene–environment interplay that underpin genetic influence on environmental measures may be developmentally important in their own right (Rutter, 2007). By actively studying these processes, researchers are able to pose more nuanced questions about how genes and environments contribute interdependently to developmental change.

### Stability and change in the home environment between childhood and adolescence

The transitional period between childhood and adolescence is a time of particularly intense physiological, psychosocial and emotional change (Simmons, Burgeson, Carlton‐Ford & Blyth, 1987; L.P. Spear, 2000; Steinberg, 2005). Underpinned, biologically, by the onset of puberty and accompanying hormonal changes and, socially, by an increase in autonomy and peer involvement, this period also sees changes in the role of an individual's home environment in their life (Laursen & Collins, 2009; Laursen, Coy & Collins, 1998; Paikoff & Brooks‐Gunn, 1991; Steinberg, 2000). When considering bidirectionality in the relationship between the developing individual and their home environment, we could conceive of this period as a likely time for an increase in child‐driven processes, with individuals increasingly inclined and able to exert an influence on the other members of their household through their behaviour. Equally, we could envision the transition into adolescence as a time when parents may seek to impose new rules or disciplinary approaches, or even to consciously reduce their influence in their children's lives. Furthermore, just as changes in both parent‐ and child‐driven processes can underpin change in the home environment across this period of development, so too can their stability dictate which aspects of it remain consistent. If parents’ values and views about parenting are fixed and stable, the nature of their influence on the home environment should not change as the child enters adolescence. Similarly, stable aspects of the child's temperament or personality might mean that some child‐driven processes endure right across development. These are empirical questions – and they can be addressed, at least in part, by examining the developmental aetiology of aspects of the home environment across this period.

Longitudinal, genetically sensitive designs allow for an examination of genetic and environmental influences and their interplay within a developmental context (Jaffee, Hanscombe, Haworth, Davis & Plomin, 2012). When the same phenotype is measured on multiple occasions, correlations between measurement occasions indicate the phenotypic stability of the trait or behaviour under study. For example, in the case of parental discipline, this would represent the extent to which individual differences in the way children experience discipline from their parents are stable across development. To the extent that variance in a variable is *un*correlated with earlier occasions of measurement, it can be considered as novel – i.e. individual differences in parental discipline at a given age that are unrelated to those in play earlier in development. In a genetically informative (e.g. twin) sample, the genetic and environmental components of both stable and age‐specific variance can be ascertained.

In child‐based twin studies, genetic influence on environmental variables is indicative of child‐driven processes. When these processes operate stably across development, this manifests as genetic influence on phenotypic stability. For the parental discipline example introduced above, this could indicate the same child behaviours and characteristics influencing the discipline he or she receives at different ages. Conversely, if *different* genes are involved at different ages, this manifests as genetic influence on phenotypic change. For parental discipline, this could indicate that child‐driven processes are operating to influence parents’ disciplinary strategies via behavioural *change* at different ages.

### Parent‐ and child‐driven processes in early adolescence: dynamic or stable?

To date, only two studies have utilized longitudinal behavioural genetic designs to investigate the role of parent and child effects in shaping the home environment across early adolescent development. Both made use of data from the Minnesota Twin Family Study (MTFS) to examine the changing aetiology of the parent–child relationship between the ages of 11 and 17. The first found that the importance of child genetic factors in explaining variance in the parent–child relationship increased between the ages of 11 and 14 (McGue, Elkins, Walden & Iacono, 2005). The second study applied growth curve models to these data (incorporating additional data from age 17), and found that initial genetic factors were strongly associated with genetic influences on change over time (Ludeke, Johnson, McGue & Iacono, 2013). These findings can be interpreted as indicating a consistent role for child‐driven processes at these ages. However, further investigation of the transitional period between childhood and adolescence is needed to ascertain whether these processes are the same as those operating earlier in development. Furthermore, the incorporation of different measures of parenting and the home environment will allow for comparative consideration of the developmental role of parent‐ and child‐driven processes in shaping the home environment at these ages.

### Present study

In the present study, we compared the developmental aetiologies of three different measures of aspects of the home environment often considered important to child and adolescent development: parents’ discipline of their child, parents’ feelings towards their child, and household chaos. Specifically, we used structural equation models to derive estimates of genetic and environmental influences on stable and age‐specific variance in children's reports on these measures, collected between the ages of 9 and 16 years. Where genetic and environmental factors from early waves continued to explain variance in the home environment throughout development, this would indicate contributions to stability across the measured period of childhood and adolescence. Conversely, evidence of new factors at later waves of measurement would indicate a developmentally dynamic mode of aetiological influence driving phenotypic change.

Overall, the primary aim of this study was to examine the respective influences of parent‐ and child‐driven processes in the home environment across late childhood and into adolescence. The isolation of child‐driven processes is possible because of the fact that, in models applied to data from children as twins, only the influences of the *child's* genes appear as genetic factors (Klahr & Burt, 2014). It is this facet of the design of the current study that allows parent‐driven processes and other factors that influence children's perceptions of their home environments to be differentiated from child‐driven processes, and their relative influence to be compared.

## Method

### Sample and procedure

The sample used in this study was taken from the ongoing Twins Early Development Study (TEDS; Haworth, Davis & Plomin, 2013). TEDS is a UK‐representative, population‐based, longitudinal study of more than 10,000 twin pairs born in England and Wales between 1994 and 1996. The zygosity of twins was determined by parent ratings of similarity, which yielded 95% accuracy when compared to zygosity as determined by DNA markers (Price, Freeman & Craig, 2000). DNA testing was also used to confirm zygosity in ambiguous cases. Data collection was performed with the informed consent of all participants. The project has ethical approval from the Institute of Psychiatry Ethics Committee.

The study sample consisted of those TEDS families who provided data via postal and online questionnaires at the 9‐, 12‐, 14‐ and 16‐year data collection waves. The overall sample size (*N *=* *13,292) includes all individuals who contributed data to the main analyses. Within this number, sample sizes vary by wave and measure and are presented in Table 1. Only two of the three birth cohorts into which the TEDS sample is divided (based upon year of birth between 1994 and 1996) were contacted at the age 9 data collection wave. This is the reason for the increase in sample size between the 9‐ and 12‐year waves, with all three cohorts being studied at the 12‐, 14‐ and 16‐year waves. In all, 3330 individuals had data available at each wave of the study, with a further 2018 having data available at all the three‐cohort waves (12‐, 14‐ and 16‐year). The sample was 48.7% male, and 92.8% participants were white European. Monozygotic (MZ) twins made up 34.8% of the sample. Attrition in TEDS is significantly associated with lower SES, non‐white ethnicity, dizygotic (DZ) zygosity and male gender, although the effects are small and the sample remains broadly representative of the population (Haworth *et al*., 2013). Of the current study sample, 3.8% were subject to medical exclusion for at least one of the four waves of data collection, with a further 3.6% excluded for other reasons, such as lack of zygosity information.

**Table 1:** Descriptive statistics and sample sizes for raw and (standardized) transformed dat*a*

Variable	Age		Mean	*SD*	Skew	Kurtosis	N (MZ)	N (DZ)
CHAOS	9	Raw	4.46	2.32	0.4	−0.14		
		**Transformed**			−**0.18**	−**0.15**	**2228**	**3746**
	12	Raw	3.99	2.04	0.48	0.25		
		**Transformed**			−**0.03**	−**0.07**	**3908**	**6878**
	14	Raw	3.75	1.95	0.52	0.22		
		**Transformed**			**0.03**	−**0.13**	**2352**	**3756**
	16	Raw	4.08	2.03	0.52	0.32		
		**Transformed**			**0.02**	−**0.06**	**1864**	**2920**
Parental discipline	9	Raw	3.19	1.57	0.37	−0.21		
		**Transformed**			−**0.13**	−**0.23**	**2180**	**3622**
	12	Raw	3.12	1.49	0.41	0.05		
		**Transformed**			−**0.14**	**0**	**3952**	**6930**
	14	Raw	3.04	1.43	0.31	0.11		
		**Transformed**			−**0.26**	**0.15**	**2298**	**3678**
	16	Raw	3.08	1.36	0.39	0.48		
		**Transformed**			−**0.22**	**0.4**	**1164**	**1718**
Parental feelings	9	Raw	4.34	2.24	0.41	0.1		
		**Transformed**			−**0.22**	**0.04**	**2080**	**3598**
	12	Raw	3.72	2.43	0.66	0.25		
		**Transformed**			**0.15**	−**0.3**	**3896**	**6822**
	14	Raw	3.66	2.66	0.84	0.52		
		**Transformed**			**0.29**	−**0.37**	**2344**	**3742**

Note

*N* = number of individuals (not twin pairs).

### Measures

#### Household chaos

Perceived levels of household chaos were assessed using a shortened version of the Confusion, Hubbub and Order Scale (CHAOS; Matheny, Wachs, Ludwig & Phillips, 1995). The original scale, assessing the levels of noise and confusion and the degree of routine in the household, consists of 11 items and has been shown to demonstrate high internal consistency (Cronbach's *α *= .79; Dumas & Nissley, 2005). The number of items was reduced to six in the short form of the questionnaire used in this study. Children were asked to respond ‘*Certainly true*’, ‘*Somewhat true*’ or ‘*Not true*’ on the following items (items 4–6 in this list are reverse‐scored): ‘You can't hear yourself think in our home’; ‘It's a real zoo in our home’; ‘There is usually a television turned on somewhere in our home’; ‘I have a regular bedtime routine’; ‘We are usually able to stay on top of things’; ‘The atmosphere in our house is calm’. The internal consistency for this measure in our study was moderate and similar across all ages (Cronbach's *α* range = .55–.59). The shortened CHAOS was administered at all four waves of data collection.

#### Parental discipline

Parental discipline was assessed via children's responses on a four‐item measure adapted from the parenting domain of a semi‐structured interview (see Deater‐Deckard, Dodge, Bates & Pettit, 1998). On a three‐point scale (‘*Not true*’; ‘*Quite true*’; ‘*Very true*’), children rated the frequency with which parents employed four different disciplinary strategies (smacking/slapping; telling off/shouting; explaining; being firm/calm) to deal with their misbehaviour. Two items were reverse‐scored to allow higher mean scores on the scale to reflect harsher parenting. On average across all ages, the internal consistency for this measure in our study was moderate (Cronbach's *α* range = .38–.46). Parental discipline was assessed by child‐report at all four waves of data collection.

#### Parental feelings

Children's perceptions of their parents’ feelings towards them were assessed using a shortened, seven‐item version of the Parental Feelings Questionnaire (PFQ; Deater‐Deckard & O'Connor, 2000). The original, 31‐item scale consisted of positivity and negativity sub‐scales, each with good internal reliability (Cronbach's *α *= .84/.90, respectively) and which are substantially negatively correlated with one another (*r *= −.61; Deater‐Deckard, Smith, Ivy & Petrill, 2005). In the shortened version used in this study, three positive items (‘I feel happy about my relationship with my Mum/Dad’; ‘My Mum/Dad finds me funny – I make him/her laugh’; ‘I feel close to my Mum/Dad’) are retained alongside four negative items (‘My Mum/Dad gets impatient with me’; ‘My Mum/Dad sometimes wishes I would leave him/her alone for a few minutes’; ‘I make my Mum/Dad angry’; ‘I make my Mum/Dad feel frustrated’). Participants again responded with ‘*Not true*’, ‘*Quite true*’ or ‘*Very true*’ and, after reverse‐scoring of the three positive items, higher scores on this measure represented more negative feelings. On average across all ages, the internal consistency for this measure in our study was good (Cronbach's *α* range = .63–.75). This variable was assessed at the 9‐, 12‐ and 14‐year waves of data collection only.

### Statistical analyses

#### Data preparation

All data were regressed on age and sex to ensure that twin correlations were not artificially inflated by twins being the same age and sex (McGue & Bouchard, 1984). Residuals were then square‐root transformed – in order to approximate normal distributions required for genetic analyses – and standardized. Descriptive statistics for the raw and transformed variables are presented in the results section.

#### Genetic analyses

The classical twin method uses information from MZ and DZ twin pairs to estimate the relative importance of additive genetic influences (A), shared environmental influences (C) and non‐shared environmental influences (E) in contributing to variation in a trait. MZ and DZ twin pairs differ in terms of their genetic relatedness, sharing 100% of their genetic material and 50% of their segregating genetic material, respectively, but sharing their rearing environment to the same extent. Consequently, additive genetic influences on a phenotype make MZ twins more similar to one another (relative to DZ twins), whereas shared environmental influences make both types of twins more similar to one another (to the same extent). Accordingly, non‐shared environmental influences incorporate anything that makes twins in the same family different from one another – including measurement error. Structural equation modelling (SEM) of the variance (within‐twin) and co‐variance (across‐twin) of a given phenotype allows for estimates of each of the variance components to be produced based on this information (Rijsdijk & Sham, 2002).

**Figure 1:**
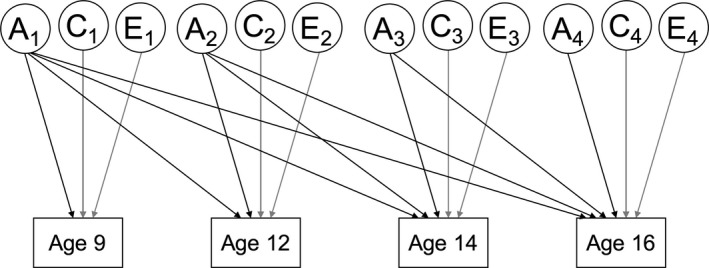
Longitudinal model: Cholesky decomposition across four waves. Note: A = genetic factors; C = shared environmental factors; E = non‐shared environmental factors; Diagonal paths – shown here for A only – estimated for all variance components.

Longitudinal analyses employed a multivariate Cholesky decomposition, wherein phenotypic variances are decomposed into genetic, shared environmental and non‐shared environmental components for each observed variable. These variance components are then allowed to influence all subsequent variables in the model. As a result, the first observed variable is influenced only by its own variance components, the second by the variance components of the first and its own novel variance components, and so on (see Figure 1). The Cholesky decomposition is well suited to the analysis of longitudinal data, as the order of variables is predetermined by their chronology. The influence of, for example, genetic factors from age 9 on variance in the same trait at later waves is interpretable as a genetic contribution to phenotypic stability.

**Figure 2:**
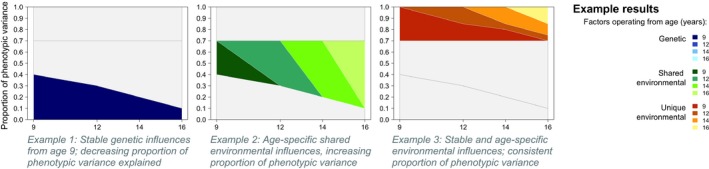
Simulated results for a single variable, with different patterns of stability and change illustrated for each aetiological component.

Figure 2 provides an example of how the results of such analyses could appear. In the simulated results displayed in the figure, a different developmental pattern is shown in each component (genetic, shared environmental and non‐shared environmental) of variance of a single variable measured at four ages (9, 12, 14 and 16 years):

1. Stable genetic influences from age 9, explaining a decreasing proportion of phenotypic variance across time, with no evidence of new genetic factors emerging (Figure 2, left panel);
2. Age‐specific shared environmental influences at all waves, explaining an increasing proportion of phenotypic variance across time, with no evidence of stability (Figure 2, centre panel);
3. Non‐shared environmental factors contributing to both change and stability and explaining a consistent proportion of variance across time (Figure 2, right panel).

It is important to note that different patterns are assigned to each variance component here for illustrative purposes only; in reality, any such patterns (or a combination) could occur for any of the variance components.

A scalar was included in models to account for sex differences in variance. This allows the overall magnitude of variance to differ between the sexes, while constraining the proportion of variance attributable to genetic and environmental parameters to be equal for males and females. All model fitting analyses were carried out in R using OpenMx (Boker, Neale, Maes, Wilde, Spiegel *et al*., 2011). OpenMx uses full‐information maximum likelihood (FIML) to estimate model parameters from raw data. The use of FIML reduces the impact of bias from selective attrition in the sample by avoiding list‐wise deletion (Enders & Bandalos, 2001).

## Results

### Summary of the main data

Table 1 shows descriptive statistics and the number of twin pairs for whom data was available for each scale, at each age. Means and standard deviations are shown for raw data only as, by definition, they are equal to 0 and 1, respectively, for standardized variables. Mean change over time was generally small, but included significant (*p *<* *.001) decreases in: household chaos at ages 12 and 14 (followed by a significant increase at 16); harsh parental discipline between 9 and 16; and negative parental feelings between 9 and 12. Some evidence of selective attrition was seen for CHAOS scores, as mean scores at age 9 were higher among those who did not provide data at 16 (*M *=* *4.67, *SD* = 2.35) than for those who provided data at all waves (*M *=* *4.37, *SD* = 2.30). There was no evidence of selective attrition for either parenting variable.

### Phenotypic analyses

The within‐variable, across‐time phenotypic correlations are presented in Table 2. Correlations between variables at adjacent ages (e.g. 9–12) were invariably larger than those across a lengthier interval (e.g. 9–16). Longitudinal correlations were generally moderate in magnitude (range for adjacent waves: .32–.55), meaning that, overall, more phenotypic change than stability was evident in the data.

**Table 2:** Within‐variable, across‐time phenotypic correlations for all variables

Age	CHAOS			Parental Discipline			Parental Feelings	
	9	12	14	9	12	14	9	12
12	0.42			0.32			0.40	
14	0.37	0.49		0.25	0.38		0.33	0.44
16	0.33	0.43	0.55	0.17	0.30	0.48	‐	‐

### Twin correlations

Table 3 presents the MZ and DZ twin correlations for all variables. MZ correlations were consistently higher than DZ correlations, indicating an aetiological role for additive genetic factors in all cases. However, DZ correlations tended to be greater than half the magnitude of MZ correlations, indicating shared environment effects making twins more similar to one another. This is consistent with the fact that the variables under study are measures of the home environment.

**Table 3:** Within‐time MZ and DZ twin correlations

	Age	MZ	DZ
CHAOS	9	0.67	0.57
	12	0.65	0.60
	14	0.58	0.50
	16	0.55	0.50
Parental discipline	9	0.54	0.43
	12	0.49	0.41
	14	0.48	0.36
	16	0.46	0.31
Parental feelings	9	0.59	0.49
	12	0.52	0.40
	14	0.54	0.36

### Genetic analyses

Longitudinal genetic analyses were undertaken to examine the relative contributions of additive genetic, shared environmental and non‐shared environmental factors to the phenotypic stability (indexed by the correlations in Table 2). Standardized, squared path estimates were produced using longitudinal Cholesky models for all variables and are presented, along with their 95% confidence intervals, in Table 4. These results are described, by variable, in the text below. In the table, genetic, shared environmental and non‐shared environmental influences factors are grouped by the age at which they originate within the model. Factors that emerge at one age and then are significant at later ages (i.e. when reading down a column) indicate contributions to phenotypic stability (as indexed by the correlations in Table 2). The within‐time influences of novel factors emerging at later waves can be interpreted as contributions to change in the phenotype.

**Table 4:** Standardized, squared path estimates (and 95% confidence intervals) from longitudinal genetic analyses

Variable	Age	9			12			14			16		
		A	C	E	A	C	E	A	C	E	A	C	E
CHAOS	9	.24	.42	34									
		(.16‐32)	(35‐.48)	(31‐37)									
	12	.01	.39	.00	.15	.12	.35						
		(.00‐.05)	(30‐.51)	(.00‐.00)	(.09‐.21)	(.01‐.22)	(33‐37)						
	14	.01	.27	.00	.02	.03	.01	.10	.15	.42			
		(.00‐.06)	(.18‐39)	(.00‐.00)	(.00‐.09)	(.00‐.15)	(.01‐.02)	(.01‐.18)	(.06‐.22)	(39‐.46)			
	16	.01	.21	.00	.02	.04	.00	.00	.14	.03	.10	.07	.41
		(.00‐.05)	(.13‐31)	(.00‐.00)	(.00‐.09)	(.00‐.17)	(.00‐.01)	(.00‐.09)	(.04‐.28)	(.01‐.05)	(.00‐.19)	(.00‐.17)	(38‐.45)
Parental Discipline	9	.23	.29	.48									
		(.13‐34)	(.21‐37)	(.44‐.52)									
	12	.03	.16	.00	.20	.10	.51						
		(.00‐.12)	(.08‐.26)	(.00‐.01)	(.09‐.28)	(.01‐.19)	(.48‐.54)						
	14	.12	.03	.00	.03	.16	.01	.08	.05	.53			
		(.02‐.29)	(.00‐.10)	(.00‐.00)	(.00‐.12)	(.02‐.29)	(.00‐.02)	(.00‐.22)	(.00‐.18)	(.49‐.57)			
	16	.04	.02	.00	.02	.08	.01	.03	.02	.05	.23	.00	.51
		(.00‐.19)	(.00‐.08)	(.00‐.00)	(.00‐.12)	(.00‐.20)	(.00‐.02)	(.00‐34)	(.00‐.13)	(.03‐.08)	(.00‐.28)	(.00‐.04)	(.46‐.57)
Parental Feelings	9	38	.20	.42									
		.28‐.48)	(.12‐.28)	(38‐.45)									
	12	.11	.13	.00	.22	.08	.47						
		(.04‐.21)	(.05‐.23)	(.00‐.01)	(.11‐32)	(.00‐.16)	(.44‐.50)						
	14	.06	.12	.00	.10	.00	.01	.27	.00	.44			
		(.02‐.15)	(.04‐.20)	(.00‐.01)	(.03‐.23)	(.00‐.08)	(.01‐.02)	(.15‐32)	(.00‐.07)	(.41‐.47)			

#### CHAOS

The proportion of variance in CHAOS explained by different genetic, shared and non‐shared environmental factors at – and across – each of the four ages is presented in the top section of Table 4. Genetic factors originating at age 9 explained 24% of the variance at age 9, but were not significant in explaining variance at any of the later ages. In contrast, age 9 shared environmental factors *were* found to influence phenotypic stability in CHAOS. These factors accounted for 42% of the variance at age 9 and remained important at all subsequent waves: age 12 (39%); age 14 (27%); and age 16 (21%). Non‐shared environmental factors from age 9 exclusively explained within‐time variance (34%), with no evident contributions to stability over time. As well as being influenced by shared environmental factors from age 9, CHAOS scores at age 12 were influenced by significant new genetic (15%), shared environmental (12%) and non‐shared environmental factors (35%). None of the new aetiological factors that came online at age 12 explained significant (or, in the case of non‐shared environmental factors, >1%) variance at any later ages. Variance in CHAOS scores at age 14 that was unexplained by earlier factors was accounted for by significant, novel aetiological factors of each type (A = 10%; C = 15%; E = 42%). Of these, shared and non‐shared environmental factors each contributed to phenotypic stability by explaining 14% and 3% of the variance at age 16. Only non‐shared environmental factors were significant in explaining age‐specific variance at 16 (41%).

**Figure 3:**
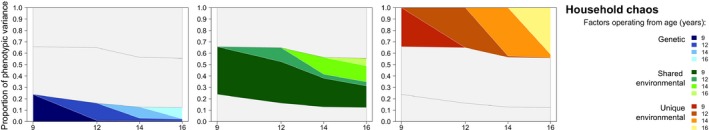
Genetic, shared environmental and non‐shared environmental influences on stability and change in child‐reported CHAOS.

The results for household chaos at all waves are illustrated graphically in Figure 3. This figure (as subsequent figures) shows the composition – in terms of the age at which constituent factors first emerged – of genetic, shared environmental and non‐shared environmental contributions to variance at each age. Contributions to stability are indicated by early factors (darker shading) continuing to account for a proportion of the phenotypic variance at later ages. New factors coming online at later ages are represented by areas of lighter shading. The proportion of variance accounted for by all factors stacked together at a given age is equivalent to a cross‐sectional estimate of genetic, shared environmental and non‐shared environmental influences at this age.

#### Parental discipline

**Figure 4:**
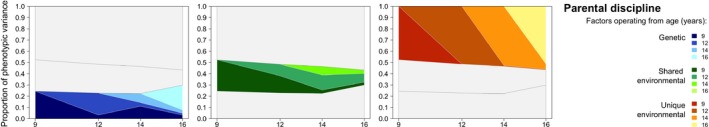
Genetic, shared environmental and non‐shared environmental influences on stability and change in child‐reported parental discipline.

Aetiological influences on change and stability in children's perceptions of parental discipline are also shown in Table 4. Age 9 genetic factors accounted for 23% of the within‐time variance but were only significant at one later age (age 14: 12% variance explained). Age 9 shared environmental factors were also significant in explaining within‐time variance in parental discipline (29%) and variance at one later age (age 12: 16%). Low phenotypic stability in this variable meant that most of the variance at later ages was explained by novel factors coming online. At 12, all age‐specific factors made significant contributions to variance (A = 20%; C = 10%; E = 51%). Age 12 shared environmental factors continued to explain variance at age 14 (16%) and thus influenced phenotypic stability across this period. Remaining variance in parental discipline at age 14 was explained by new aetiological factors coming online; of these, only non‐shared environmental factors (53%) reached significance. Variance in the parental discipline measure at age 16 was also significantly accounted for by non‐shared environmental influences. Predominantly, these originated at 16 (explaining 51% variance), but age 14 non‐shared environmental influences also accounted for 5% variance at this age. Figure 4 summarizes the results from the longitudinal models for this variable.

#### Parental feelings

**Figure 5:**
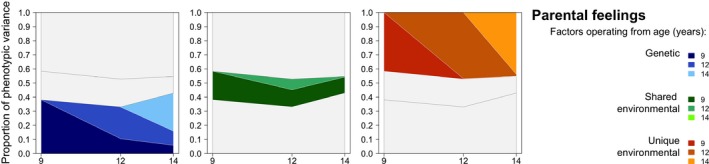
Genetic, shared environmental and non‐shared environmental influences on stability and change in child‐reported parental feelings.

Substantial overall influence of genetic factors on variance in the parental feelings variable contributed to both stability and change. Genetic factors from age 9, which explained 38% of the variance at that age, also continued to influence children's reports of parental feelings significantly at ages 12 and 14 (11% and 6% of the variance accounted for; Table 4). Age 9 shared environmental factors were also a source of stability, accounting for 20% within‐time variance and a further 13% and 12% of the variance at respective subsequent waves. Age 9 non‐shared environmental factors were again significant, substantial and entirely age‐specific, accounting for 42% of the within‐time variance only. Novel genetic and non‐shared environmental factors at age 12 explained, respectively, 22% and 47% of the variance in parental feelings at this age, while age 12 shared environmental factors were not significant. Genetic factors at age 12 explained 10% of the variance at age 14 (as well as 1% from age 12 non‐shared environmental factors). All remaining variance in parental feelings at age 14 was accounted for by novel, age‐specific genetic (27%) and non‐shared environmental influences (44%). These results are shown graphically in Figure 5.

## Discussion

Ours is the first longitudinal, genetically informative study to examine stability and change in different measures of the home environment across childhood and adolescence. We found evidence of child‐driven processes underpinning change in measures of the home environment during this transitional period of development. In contrast, parent‐driven processes and family‐wide factors were the main source of stability in child reports of parenting and household chaos at this time. This apparent difference in the developmental structure of child‐driven *versus* parent‐driven processes in shaping the home environment has several implications for developmental theory and research into early adolescent development. These are discussed in detail below.

### Stability and change in the home environment

We observed moderate stability in children's reports of parenting and household chaos across the period of study, from late childhood to early adolescence. Some continuity within the home environment across development is expected, even during the transition to adolescence, and is typically understood in relation to the stability of attachment, interdependence and communication patterns between parents and children (Laursen & Collins, 2009). Estimates of stability, given that they are derived from the phenotypic correlations between individuals’ scores on the same scale at different measurement occasions, are necessarily subject to test–retest reliability. This means that a portion of the discontinuity observed here is likely due to occasion‐specific measurement error (see Limitations section below for a further discussion of this point). Nonetheless, a finding of substantial discontinuity in the home environment at this period of development is also in line with evidence from previous work in this area (Laursen & Collins, 2009; Laursen *et al*., 1998; Paikoff & Brooks‐Gunn, 1991; Steinberg, 2000). In order to begin to interpret these patterns of phenotypic stability and change in terms of underlying bidirectional processes, we now turn to a discussion of the aetiological results.

### Genetic influences on stability and change in measures of the home environment

Child genetic factors were found predominantly to drive change, rather than stability, in all measures of the home environment. The contribution of genetic factors primarily to change in our home environmental variables is in stark contrast to the findings regarding influences on stability and change in emotional and behavioural development. These studies have primarily shown genetic factors to account for stability and environmental factors to account for change (e.g. Haberstick, Schmitz, Young & Hewitt, 2005; Hoekstra, Bartels, Hudziak, Van Beijsterveldt & Boomsma, 2008; Trzaskowski, Zavos, Haworth, Plomin & Eley, 2012; Waszczuk, Zavos & Eley, 2013; see Hannigan, Walaker, Waszczuk, McAdams & Eley, 2016). The influence of different genetic factors across development has been shown in some behavioural studies, and conceptualized as evidence of a ‘developmentally dynamic genome’ (e.g. Kendler, Gardner & Lichtenstein, 2008; Kendler, Gardner, Annas, Michael, Eaves *et al*., 2008; van Beijsterveldt, Bartels, Hudziak & Boomsma, 2003; Zavos, Gregory & Eley, 2012). Nonetheless, our finding of genetic influence on change consistently *exceeding* that on stability appears to be a characteristic of these environmental variables that differs from commonly studied behavioural and cognitive phenotypes.

The broad discrepancy between these results and the results of studies of behavioural phenotypes is particularly striking given the typical interpretation of genetic influence on measures of the environment in child‐based designs: as the effect of children's genetically influenced *behaviour* shaping aspects of their home environment (Avinun & Knafo, 2014; Plomin, Loehlin & DeFries, 1985; Scarr & McCartney, 1983). The implication of our finding, that these effects of child behaviour are developmentally dynamic, is that different genes are influencing environment‐shaping behaviours at different stages of the transition to adolescence. For example, hormonal changes during puberty may result in prominent new behavioural characteristics that evoke entirely different responses from caregivers or siblings from those evoked earlier in development. Given the relative lack of genetic stability in our variables, this possibility that *change* in children's behaviour shapes their home environment in a way that stability does not is intriguing and worthy of further investigation.

As children develop, their capacity to influence characteristics of their environments through their behaviour increases (Scarr & McCartney, 1983). For example, an older child's ability to engage in meaningful interactions with – and evoke responses from – parents and other caregivers will be greater than that of a prelinguistic infant. Similarly, adolescents are likely to experience increasing levels of independence and be able to exert more control over when, how and with whom they spend their time in the household (Beyers & Goossens, 1999; H.J. Spear & Kulbok, 2004; Steinberg & Silverberg, 1986). Change in the ways in which developing individuals can shape their environment has been posited as a potential explanation for the overall increases in genetic influence seen for many behavioural traits in adolescence (Bergen, Gardner & Kendler, 2007; Hanscombe, Haworth, Davis, Jaffee & Plomin, 2010; Rice, Harold & Thapar, 2003). Of note, the ‘genetic amplification’, in which stable genetic influences account for increasing proportions of the variance across time, that was identified in a previous longitudinal study of the aetiology of the parent–child relationship (Ludeke *et al*., 2013) was largely absent in our sample.

A potential alternative interpretation of the finding of widespread genetic influence on change across development must also be considered. This is that genetic factors influenced children's scores on measures of the home environment via the perceptual (or subjective) element of their reporting. If this was the case, new genetic influences on our home environmental variables at ages 12, 14 and 16 could relate to developmental changes in the way in which children perceived their environment, rather than in the environment per se. For example, increasing exposure to a wider range of social experiences may, in tandem with developing social cognition abilities, result in children recruiting different perceptual processes in their appraisal of their home environment (Blakemore & Choudhury, 2006; Eccles, Midgley, Wigfield, Buchanan, Reuman *et al*., 1993).

These two mechanisms by which children's genes may influence their scores on measures of the home environment – behaviour and perception – are not mutually exclusive. Indeed, as far as theoretical approaches to child development are concerned, both fall into the category child‐driven processes. Certainly, neither represents a model of direct environmental influence on the individual as a passive recipient. Instead, they relate to the engagement – behavioural or perceptual – of the individual with the environment around them. As such, either may relate functionally to the developmental outcomes with which such environmental phenotypes are often associated. Future work could incorporate observer and/or parent reports into the developmental genetic design in order to attempt to distinguish behavioural and perceptual effects in this regard. Alternatively, this question could be addressed by comparing the aetiological overlap between aspects of the home environment and measures of both behaviour and perception longitudinally.

Before moving to discuss the results for environmental factors, it is worth noting that despite the consistency of the pattern of genetic factors predominantly driving change rather than stability across the variables in the study, we did find differences in the overall magnitude of genetic influence on each variable. Genetic factors were most influential for parental feelings (explaining, on average, 38% variance at each age), slightly less for parental discipline (≈25% variance explained) and less again for household chaos (≈17%). To some extent, this aligns with expectations for these variables, in terms of child‐driven effects. In the case of the parenting variables, it has been shown previously that parents’ feelings towards their children are more influenced by children's genetic factors than are their disciplinary strategies (see Kendler & Baker, 2007, for a review), although a recent meta‐analysis found this difference to be significant only for parent‐reports and not child‐reports or observational data (Avinun & Knafo, 2014). The greater heritability of parental feelings is usually attributed to the fact that the emotional responses children evoke in their parents are automatic, and not subject to the parent's deliberate control (Klahr & Burt, 2014). Although the discipline a child receives may also be correlated with their genotype via this process, this aspect of parenting is thought to be more subject to parents’ conscious, strategic control, and to external factors, such as culture (Shikishima, Hiraishi, Yamagata, Neiderhiser & Ando, 2012). This difference could explain these results in the current study, with the further reduction in child‐driven effects on household chaos attributable to its focus on the wider home environment, rather than any interactions with one specific child. Despite the intuitive nature of these interpretations of differences in the magnitude of genetic influence between the variables, it is important to note that differences in the reliability of measures hampers the extent to which firm conclusions can be drawn (see the Limitations section below for further discussion on this).

### Environmental influences on stability and change in measures of the home environment

Shared environmental factors were found to be most important for driving stability in measures of the home environment, though it should be reiterated that overall stability was only moderate for these measures, with correlations between time points ranging from .32 to .55. For household chaos, the fact that shared environmental factors were seen primarily to produce stability ties in with what is known about specific influences on household characteristics that are, themselves, predominantly stable (e.g. SES, neighbourhood characteristics; Bradley, Corwyn, McAdoo & García Coll, 2001b; Dumas, Nissley, Nordstrom, Smith, Prinz *et al*., 2005). Indeed, the large influence of stable shared environmental factors is consistent with the origins of the CHAOS measure, which was intentionally designed as an index of aspects of the home environment that are largely expected to be shared by cohabitants.

Wider cultural and environmental factors may also explain the prevalence of shared environmental factors in producing stability in the parenting variables. However, stable parental characteristics, such as personality traits, are also likely to have influenced parenting, and these would also manifest as stable shared environmental influences. This is true irrespective of the fact that such characteristics of parents are likely to be genetically influenced because, in a child‐based design, only the influence of the *child's* genes load on the genetic component of the models (Klahr & Burt, 2014). The importance of parental characteristics is supported by theoretical accounts of the role of parent‐driven processes in shaping the dyadic relationship with their child, which hold that personality characteristics influence the relationship both directly and via a feedback loop relating to the parent's own exposure and sensitivity to stress and support (Belsky, 1984; Darling & Steinberg, 1993). Findings of predominant stability in adult personality (e.g. Caspi, Roberts & Shiner, 2005; Costa & McCrae, 1988, 1997) are consistent with this idea that parent‐driven processes may operate somewhat stably to bring continuity to the child's home environment across development.

As well as stability, there was some indication of change in shared environmental influences; in particular, for parental discipline. This could be interpreted as evidence that parents, at least to some degree, seek to employ age‐specific (rather than child‐specific) disciplinary strategies. Even if this is the case, the greater magnitude of non‐shared environmental influences at each wave indicates that children experience these strategies somewhat differentially. The consequences of this kind of discrepancy, in terms of associations between (actual or perceived) differential parenting and developmental outcomes, have not yet been fully explored (e.g. Burt, McGue, Iacono & Krueger, 2006; Feinberg & Hetherington, 2001; Mullineaux, Deater‐Deckard, Petrill & Thompson, 2009; Reiss, Hetherington, Plomin, Howe, Simmens *et al*., 1995). Parent‐driven processes remain plausible explanations for such associations, as has been shown elsewhere (e.g. Bornovalova, Cummings, Hunt, Blazei, Malone *et al*., 2014; Narusyte, Neiderhiser, Andershed, D'Onofrio, Reiss *et al*., 2011; see Maccoby, 2000, for a review).

As indicated above, non‐shared environmental influences were significantly influential for all variables at all ages, and operated almost exclusively to drive phenotypic change. Interpretation of the results from this component of the models is hampered by confounding from occasion‐specific measurement error, which makes twins’ responses differ from one another in an unsystematic way. However, the results of previous studies indicate that some influence of ‘real’ non‐shared environmental factors is likely, and may be related to children spending increasing amounts of time outside of the household (Mullineaux *et al*., 2009; Pike, Reiss, Hetherington & Plomin, 1996; Turkheimer & Waldron, 2000). Measurement error is thought to reduce in adolescence as children become more reliable reporters (Ludeke *et al*., 2013) and it is noteworthy that, in the present study, wave‐to‐wave stability increased with age for each variable (see Table 2). This is consistent with the presence of some ‘real’ non‐shared environmental influences. Nonetheless, the true magnitude of the role of non‐shared environmental influences in producing differences in children's perceptions of their home environment cannot be determined here. Our interpretation of these results is therefore constrained to the non‐trivial observation that non‐shared environmental influences are not a source of stability in the children's perceptions of their home environments during the transition from childhood to early adolescence.

## Limitations

As well as sharing all limitations and assumptions that are inherent to the twin design, such as the now well‐explored equal environments assumption (e.g. Derks, Dolan & Boomsma, 2006), this study had some specific limitations. First, the exclusive use of child‐report data means that results are most informative as to children's *perceptions* of household chaos and parenting. Although these perceptions could be qualitatively different from, for example, parents’ perceptions of the same phenotypes, they are, conceivably, of particular importance in developmental terms. This is because, whether a child's report on their home environment represents an accurate reflection or a subjective perception, it will likely reflect their developmental experience. Nonetheless, this facet of the design prohibits us from being able to disentangle the respective roles of children's behaviour and their perception in producing genetic influence on the environmental variables, and this is a limitation. Quantifying the relative objectivity and subjectivity of these reports would therefore be beneficial, and further work using data from multiple raters could enable this. Furthermore, other study designs, such as the children‐of‐twins design (in which parents are twins), might be also well placed to investigate these phenotypes from the parent perspective (McAdams, Neiderhiser, Rijsdijk, Narusyte, Lichtenstein *et al*., 2014).

A second, related limitation concerns the relationship between measurement reliability and the non‐shared environmental contributions of variance estimated in the models. Internal consistencies (Cronbach's α) were generally lower than is considered satisfactory in the development of psychometric measures, with only the parental feelings scale consistently reaching this level. This coefficient relates to the clustering of items within scales, so a low coefficient may relate to the breadth of the construct being assessed, as well as poor reliability. However, given the imperfect reliability of our measures, it is very likely that some error of measurement was included in the data from each measure. In behavioural genetic models, measurement error loads onto non‐shared environmental estimates (because error is essentially random and so does not correlate within families). Given the pattern of non‐shared environmental influences in the current study, it is also evident that any measurement error was not correlated across time. Therefore, occasion‐specific measurement error in our study would have loaded onto estimates of non‐shared environmental contributions to change and decreased the amount of stable, ‘real’ variance available for decomposition. However, this limitation could only result in an under‐estimation of genetic or shared environmental influences and thus the results of this decomposition remain interpretable as lower bound estimates of genetic and shared environmental stability and change in the home environment. While the imperfect reliability of our measures serves as a limitation to our study, it is important to note that it cannot explain the longitudinal results. That is, measurement error would not explain the contrast between the primary pattern of results in the current study (genetic influence on change, common environmental stability) and those typically found in longitudinal genetic studies of behavioural and cognitive phenotypes (i.e. predominant genetic stability; Hannigan *et al*., 2016).

Finally, genetically informative, child‐based investigations of parenting, such as the present study, are well equipped to address genetic confounding mediated through evocative effects that associate with the child's genotype. However, the influence of passive gene–environment correlation – i.e. parents providing both genes *and* environments for children – is not quantified in such designs (Avinun & Knafo, 2014). Instead, as noted in the interpretation of the environmental results, some of the effects of parents’ genes will accumulate in the shared environmental components of models. Genetic confounding of measures of children's environments that is mediated through parents’ genes can be investigated using other behavioural genetic designs, such as the children‐of‐twins design (McAdams *et al*., 2014).

## Conclusions

The results of this study highlight the importance of considering the home environment as both a context for child development *and* a consequence of the behavioural and cognitive change that accompany it. Developmentally dynamic genetic influences on measures of the home environment across early adolescence indicate that *different* child‐driven processes may be important at *different* stages of development. In contrast, parent‐driven processes may operate to provide more developmental stability in the home environment during the transition between childhood and adolescence. We have shown longitudinal genetic models to be a powerful tool for the investigation of parent‐ and child‐driven processes across development, allowing variance in home environmental measures to be decomposed aetiologically (in terms of the genetic and environmental influences) and temporally (in terms of when these influences may first ‘come online’ and subsequently endure or wane). Understanding the dynamics of the bidirectional processes that shape the home environment developmentally is a challenge that will require the integration of methodological approaches from across the full breadth of developmental science.
